# F136. PARSING DUP TO REFINE EARLY DETECTION: QUANTILE REGRESSION OF RESULTS FROM THE SCANDINAVIAN TIPS STUDY

**DOI:** 10.1093/schbul/sby017.667

**Published:** 2018-04-01

**Authors:** Maria Ferrara, Sinan Guloksuz, Shadie Burke, Fangyong Li, Svein Friis, Wenche ten Velden Hegelstad, Inge Joa, Jan Olav Johannessen, Ingrid Melle, Erik Simonsen, Vinod Srihari

**Affiliations:** 1 Yale University, AUSL Modena; 2 Academic Hospital Maastricht; 3 Yale University; 4 Oslo University Hospital, Institute of Clinical Medicine, University of Oslo; 5 TIPS – Centre for Clinical Research in Psychosis, Stavanger University Hospital; 6 Institute of Clinical Medicine, University of Oslo, Oslo University Hospital, NORMENT KG Jebsen Centre for Psychosis Research, Oslo University Hospital; 7 University of Copenhagen

## Abstract

**Background:**

Prolonged duration of untreated psychosis (DUP) is associated with poor outcome. The Scandinavian TIPS study deployed an early detection (ED) campaign to halve DUP. However, while reducing DUP will improve outcomes for most patients, there are some for whom prolonged DUP is a byproduct of an insidiously illness rather than a modifiable prognostic factor. It is also unclear whether the success of an ED program relies on targeting those with longer or shorter DUP. Previously, we demonstrated that quantile regression (QR) can both manage skewed distributions and allow analysis for meaningful heterogeneity in DUP. The current study aims to investigate the utility of QR to analyze the impact of an ED campaign across the DUP distribution, using data from the TIPS study. We hypothesized the effectiveness of TIPS’s ED campaign will vary across different quantiles of DUP.

**Methods:**

Between 1997 and 2000, a comprehensive early detection (ED) program with public information campaigns and low-threshold psychosis detection teams was established in one health-care area (ED-area), but not in a comparable area (No-ED area). Users with DSM IV non-organic non-affective first episode psychosis were consecutively recruited. Demographic, social and clinical characteristics of people enrolled in an ED area were compared to those coming from a No-ED area. Quantile regression can model the relationship between conditional quantiles of response and independent variables. Unlike ordinary least-squares regression that focuses on conditional mean response, QR estimates the heterogeneous effects of ED across different quantiles of DUP, rather than presuming a uniform mean effect. It is particularly useful when the differential effect of predictors on lower or upper quantile of outcome are of interest. In this study, we examined the impact of ED across the entire quantiles of DUP, particularly on Q1, Q2, Q3, dividing data into four quartiles. A post hoc analysis of the effect of gender, marital status, premorbid adjustment social level, and social cluster on quartiles of DUP was also conducted.

**Results:**

The total sample included 301 subjects, of which 161 belonged to an early detection (ED) area. If compared to users from No-ED area, ED users were younger (mean age 25 vs 31), and mainly unmarried (80% vs 62%).

QR highlighted that ED had no effect on the first quartile (Q1) of DUP, with very short DUP, even in the No-ED area. ED was significantly associated with a reduction in the second quartile of DUP (median) (11 weeks reduction, p<0.001), and the third quartile of DUP (Q3) (41 weeks reduction, p=0.01). The effect of ED was significantly stronger on last quartile than Q1(p=0.01) and Q2 (p=0.04) suggesting a stronger effect of campaign on people with longer DUP.

After controlling for age and marital status, the ED campaign’s effect on Q3 of DUP significantly differed by gender: only male users in the ED group showed a significant reduction in this quartile of DUP (coefficient [SE] at Q3=−46.6; P = .01), suggesting an interaction between gender and ED campaigns in reducing DUP.

**Discussion:**

Quantile regression represents a powerful tool reveal the different effects of an ED campaign across DUP distribution. The upper tail of DUP benefited the most from ED program: users with longer DUP might be hesitant to engage into treatment because of a longstanding active psychosis or failed attempts in the community to receive care. Very short DUP, highly associated with rapidly escalating symptoms, was not affected by the campaign. This could represent a subgroup of patients for which no specific ED efforts are needed or further reductions may need strategies targeting prodromal signs. These findings have been fertile ground for generating hypotheses that could lead to targeted ED efforts.

